# Genetic analysis of *Octopus cyanea* reveals high gene flow in the South‐West Indian Ocean

**DOI:** 10.1002/ece3.11205

**Published:** 2024-04-04

**Authors:** Charles R. Treleven, Mary A. Kishe, Mathew O. Silas, Benjamin P. Ngatunga, Bigeyo N. Kuboja, Said S. Mgeleka, Amy L. Taylor, Megan A. M. Elsmore, Amy J. E. Healey, Warwick H. H. Sauer, Paul W. Shaw, Niall J. McKeown

**Affiliations:** ^1^ Department of Life Sciences Aberystwyth University Aberystwyth UK; ^2^ Fisheries Research Institute (TAFIRI) Dar es Salaam Tanzania; ^3^ Department of Ecology, Environment and Plant Sciences Stockholm University Stockholm Sweden; ^4^ Department of Ichthyology & Fisheries Science Rhodes University Makhanda South Africa

**Keywords:** cephalopod, connectivity, fishery, genetic structure, stock, sustainability

## Abstract

*Octopus cyanea* (Gray, 1849), abundant in the South‐West Indian Ocean (SWIO), constitutes a vital resource for both subsistence and commercial fisheries. However, despite this socioeconomic importance, and recent indications of overfishing, little is known about the population structure of *O. cyanea* in the region. To inform sustainable management strategies, this study assessed the spatio‐temporal population structure and genetic variability of *O. cyanea* at 20 sites in the SWIO (Kenya, Tanzania, Mozambique, Madagascar, Mauritius, Rodrigues, and the Seychelle Islands) by complementary analysis of mitochondrial DNA (mtDNA) noncoding region (NCR) sequences and microsatellite markers. MtDNA analysis revealed a shallow phylogeny across the region, with demographic tests suggesting historic population fluctuations that could be linked to glacial cycles. Contrary to expectations, NCR variation was comparable to other mtDNA regions, indicating that the NCR is not a hypervariable region. Both nuclear and mtDNA marker types revealed a lack of genetic structure compatible with high gene flow throughout the region. As adults are sedentary, this gene flow likely reflects connectivity by paralarval dispersal. All samples reported heterozygote deficits, which, given the overall absence of structure, likely reflect ephemeral larval recruitment variability. Levels of mtDNA and nuclear variability were similar at all locations and congruent with those previously reported for harvested Octopodidae, implying resilience to genetic erosion by drift, providing current stock sizes are maintained. However, as *O. cyanea* stocks in the SWIO represent a single, highly connected population, fisheries may benefit from additional management measures, such as rotational closures aligned with paralarval ecology and spanning geopolitical boundaries.

## Graphical abstract

Genetic data confirm high population connectivity across the SWIO for *O. cyanea*, This requires cross border management initiatives and future research and public engagement activities focusing on paralarvae ecology. There is further evidence that the mtDNA NCR does not seem to be rapidly mutating in cephalopods.

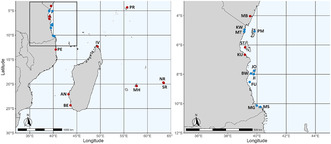

## 1 INTRODUCTION

Cephalopods are coming under increasing harvesting pressure globally in response to a decline in finfish stocks (Caddy & Rodhouse, 1998; Pauly & Zeller, 2016). Various studies have already evidenced the overexploitation of cephalopods (Guard & Mgaya, 2002; Sauer et al., 2011; Meissa & Gascuel, 2014; Rocliffe & Harris, 2015; Xavier et al., 2014), and cautioned of adverse wider ecosystem effects as many cephalopods occupy key trophic positions acting as both predator and prey (Hunsicker et al., 2010; Sabolić et al., 2021). Therefore, obtaining information on population demographics is vital for the development of sustainable management strategies (Moreira et al., 2011; Sale, 2004). Gaining such information by traditional fisheries methods in cephalopods is challenging due to their soft bodies with few hard structures, considerable phenotypic plasticity (Arkhipkin et al., 2021; McKeown, Arkhipkin, & Shaw, 2019), and sensitivity to physical tagging studies (Anderson & Babcock, 1999; Semmens et al., 2007). However, genetic methods have proven effective in identifying spatial structure and connectivity patterns in cephalopods (McKeown, Arkhipkin, & Shaw, 2019; Oosthuizen et al., 2004; Shaw et al., 1999, 2010), facilitating the delineation of meaningful biologically based management units (Coltman, 2008).

The day octopus (*Octopus cyanea* Gray, 1849) is primarily benthic, residing in the shallow coastal reefs throughout the Indo‐Pacific to the Hawaiian Islands and including the South‐West Indian Ocean (SWIO), comprising the East African coast, Madagascar, Mauritius, Seychelles, and islands in between (Chande et al., 2021; Herwig et al., 2012; Norman, 1991). The species is characterized by a short lifespan of approximately 12–15 months (Guard, 2009; Van Heukelem, 1973), rapid growth (Van Heukelem, 1973, 1983) and early sexual maturity (Guard & Mgaya, 2002). Individuals are crepuscular and inhabit conspicuous shallow water “dens” during inactive hours or while brooding, making them susceptible to artisanal fishing efforts using hand tools while inactive (Benbow et al., 2014; Sobrino et al., 2011). Females are highly fecund (100,000–400,000 eggs) and reported to be more vulnerable to capture (Forsythe & Hanlon, 1997; Guard & Mgaya, 2002). The period from the brood's initial spawning and maternal care is approximately 20–30 days; at this point, the female dies, and the paralarvae hatch and enter the water column. The subsequent planktonic paralarval phase takes another 30 days until it reaches a critical size, and thereafter, paralarvae settle and take up a benthic habit (Guard, 2009; Guard & Mgaya, 2002).

Octopus species comprise a substantial share of the dietary protein in local communities in the SWIO, while also providing a valuable commercial fishery to local fishers and nations for trade with foreign collection companies (Rocliffe & Harris, 2016). Tanzania (1241 t), Madagascar (1087 t), and Mauritius (324 t) were the major producers and exporters of octopus species in the SWIO in 2015, with other regional countries landing sizable figures (Rocliffe & Harris, 2016; Sauer et al., 2021). Within the region, *O. cyanea* is the prominent octopus species caught by artisanal fishers, comprising ~99% of the total catch of the Tanzanian fishery (Guard & Mgaya, 2002), and 95% in Madagascar (Epps, 2007). Despite octopus species life histories seemingly conferring resilience to overfishing, the continual growth in the global market and increased harvesting pressure (including unreported and illegal fishing) have coincided with a decline in fishery productivity (FAO, 2020; Guard & Mgaya, 2002).

The local socioeconomic importance of *O. cyanea* directs consideration of stock structure for the establishment of effective management strategies. A single population genetic study of *O. cyanea* in the SWIO, based on mtDNA sequence data, reported some numerically small yet statistically significant interregional differentiation (*Φ*_ST_), but the majority of pairwise *Φ*_ST_ values were nonsignificant and there was no detectable isolation by distance (IBD) effect (Van Nieuwenhove et al., 2019). Given the notorious difficulty of interpreting such weak genetic structure in terms of contemporary connectivity (Waples, 1998), particularly when based on a single locus, Van Nieuwenhove et al. (2019) highlighted the need for further studies employing more powerful nuclear markers.

The aim of the present study was to utilize both nuclear microsatellite DNA markers and the mtDNA noncoding region (NCR) to further assess the genetic variation of *O. cyanea* populations across the SWIO. Samples were collected from 20 sites spanning six countries and the autonomous territory of Rodrigues, representing both the East African continental coastline and oceanic islands. This sampling also spanned oceanographic systems that could potentially affect larval dispersal across the region. Samples were collected during two time periods (2010–12 and 2020), permitting the assessment of the temporal stability of any resolved structure, a recognized powerful approach to interpreting the biological significance of low levels of genetic structure (Waples, 1998). The inclusion of mtDNA facilitated comparison with the findings of Van Nieuwenhove et al. (2019) while also providing insight into the respective properties of the NCR, which has been largely unexplored in cephalopods.

## 2 MATERIALS AND METHODS

### 2.1 Sampling

**FIGURE 1:**
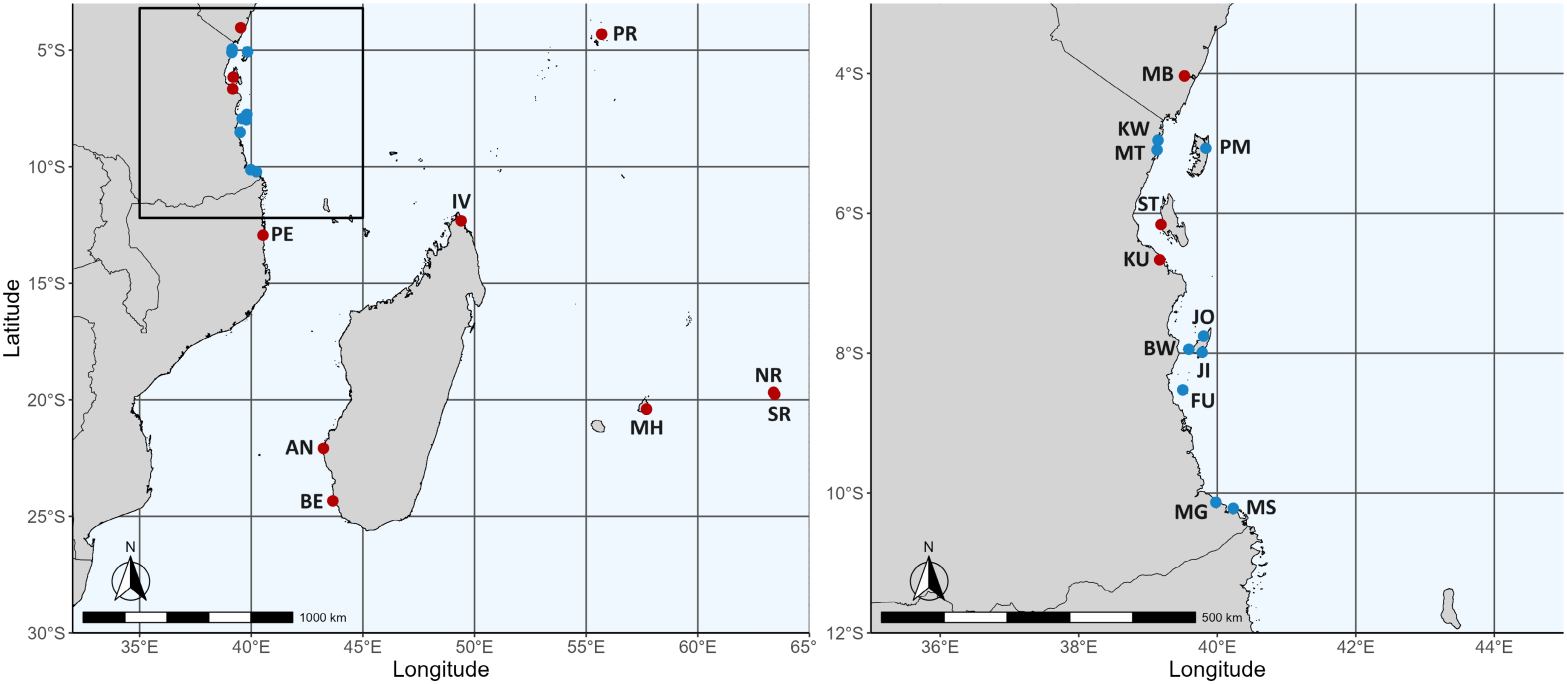
Sampling sites of *O. cyanea* in the SWIO. Red points represent sites sampled from 2010 to 2012; samples collected in 2020 are blue. AN, Andavadoaka; BE, Beheloke; BW, Bwejuu; FU, Funguni; IV, Ivovona; JI, Jibondo; JO, Jojo; KU, Kunduchi; KW, Kwale; MB, Mombasa; MG, Mgao; MH, Mahebourg; MS, Msangamkuu; MT, Mtambwe; NR, North Rodrigues; PE, Pemba; PM, Pembeni; PR, Praslin; SR, South Rodrigues; ST, Stone Town.

*Octopus cyanea* arm tips were sampled from 11 sites spanning the SWIO by artisanal fishers and direct sampling between 2010 and 2012. An additional 9 sites were sampled during a targeted survey of Tanzanian waters in 2020 (Table 1; Figure 1). All octopus arm tips were sampled from natural populations, immediately preserved in 95% ethanol, and frozen at −20°C when feasible.

**TABLE 1:** Summary information for *O. cyanea* samples from the SWIO using the mitochondrial noncoding region (*N* = 415) and 7 microsatellite markers (*N* = 962): sample size (*n*), number of haplotypes (*N*_hap_), haplotype diversity *(h*), nucleotide diversity (*π*), Fu's Fs test (*F*_S_), Tajima's D test (*D*), mean number of alleles (*N*_*A*_), rarified allelic richness (15 diploid individuals) (*A*_R_), observed heterozygosity (*H*_O_), expected heterozygosity (*H*_E_), and multilocus inbreeding coefficient values (*F*_IS_).

			Mitochondrial						Microsatellite					
State/territory and site	Code	Year	*n*	*N*_hap_	*h*	*π*	*F*_S_	*D*	*n*	*N*_A_	*A*_R_	*H*_O_	*H*_E_	*F*_IS_
Kenya														
Mombasa	MB	2010–12	19	2	0.105	0.0002	−0.838	−1.165	57	14.429	10.686	0.749	0.810	0.084*
Tanzania														
Kunduchi	KU	2010–12	14	4	0.571	0.0017	−1.118	−0.708	58	15.429	10.826	0.794	0.816	0.033
Stone Town	ST	2010–12	22	3	0.177	0.0004	−1.974**	−0.515*	78	15.429	10.834	0.779	0.832	0.066*
Bwejuu	BW	2020	18	4	0.314	0.0005	−2.603**	−1.508*	20	11.143	10.523	0.510	0.792	0.376*
Funguni	FU	2020	28	3	0.140	0.0003	−2.268**	−1.511*	30	12.143	10.511	0.602	0.816	0.278*
Jibondo	JI	2020	18	4	0.471	0.0012	−1.596*	−1.131	20	10.286	9.740	0.631	0.825	0.259*
Jojo	JO	2020	12	2	0.167	0.0004	−0.476	−1.141	17	10.286	10.089	0.607	0.788	0.258*
Kwale	KW	2020	28	3	0.204	0.0005	−1.586*	−1.241	30	12	10.395	0.661	0.793	0.182*
Mgao	MG	2020	22	3	0.177	0.0004	−1.974**	−1.515*	24	11.143	10.209	0.614	0.812	0.265*
Msangamkuu	MS	2020	26	3	0.151	0.0004	−2.176**	−1.513*	30	11.429	9.869	0.543	0.792	0.335*
Mtambwe	MT	2020	28	4	0.267	0.0006	−2.610**	−1.527*	29	11.429	10.222	0.632	0.815	0.244*
Pembeni	PM	2020	29	6	0.374	0.0009	−4.737***	−1.868**	30	13	10.998	0.629	0.855	0.279*
Mozambique														
Pemba	PE	2010–12	18	2	0.209	0.0005	−0.011	−0.529	52	15	10.873	0.730	0.830	0.127*
Madagascar														
Andavadoaka	AN	2010–12	11	4	0.491	0.0017	−1.415*	−1.712*	76	14.857	10.994	0.744	0.845	0.112*
Beheloke	BE	2010–12	23	4	0.486	0.0012	−1.306	−0.888	75	14.857	10.950	0.809	0.852	0.048*
Ivovona	IV	2010–12	8	1	NA	NA	NA	NA	54	14.857	11.010	0.769	0.827	0.080*
Mauritius														
Mahebourg	MH	2010–12	46	4	0.167	0.0004	−3.432***	−1.576*	73	15.429	10.470	0.770	0.822	0.071*
Rodrigues														
North Rodrigues	NR	2010–12	16	2	0.125	0.0003	−0.700	−1.162	40	13.714	10.982	0.734	0.819	0.114*
South Rodrigues	SR	2010–12	11	2	0.182	0.0004	−0.410	−1.129	50	13.714	10.817	0.786	0.825	0.050*
Seychelles														
Praslin	PR	2010–12	18	4	0.399	0.0025	0.062	−1.854*	106	16.429	11.075	0.786	0.840	0.072*

*Note*: Significant departure of values from expectations is indicated by *p < .05, **p < .01, and ***p < .001.

### 2.2 DNA extraction and PCR Amplification

Genomic DNA was isolated from ~20 to 25 mg of arm tip muscle tissue from 962 individuals of *O. cyanea* using the Qiagen DNeasy® blood and tissue kit following the manufacturers' purification of total DNA from animal tissues (spin‐column) protocol. All 962 individuals were Polymerase Chain Reaction (PCR) amplified at seven species‐specific microsatellite loci (ROC1, ROC6, ROC17, ROC28, ROC32, OC18, and OC22; McKeown et al., 2018). In order to make comparisons with Van Nieuwenhove et al. (2019), a fragment of mtDNA NCR was amplified for a subset of 415 individuals using species‐specific forward (F: TCCTGTTAATGGTCAGGGTCTAA) and reverse (R: GATTGGGTCTCCTCCACCTC) primers. Reaction mixes consisted of 10 μL total volumes containing 2 μL of template DNA, 2 μL of distilled water, 5 μL Biomix (Meridian Bioscience), and 0.5 μM of forward and reverse primers. The PCR thermoprofile comprised an initial denaturation step of 95°C for 180 s, followed by 35 cycles of 95°C for 30 s, a specific annealing temperature of 30 s, 72°C for 30 s, and a final extension of 72°C for 180 s. NCR amplicons were sequenced with the forward primer using BigDye technology and an ABI 3730 DNA analyzer (Applied Biosystems). Microsatellite amplicon fragment sizes were separated using the same ABI 3730 platform, and alleles were scored manually using PEAKSCANNER version 1.0 (Applied Biosystems).

### 2.3 Statistical analyses

#### 2.3.1 Analysis of mitochondrial DNA data

Sequence chromatograms were visually inspected and trimmed in Chromas version 2.6.6 (Technelysium Pty Ltd., Australia). Sequences were then aligned by ClustalW in BIOEDIT (Hall, 1999; Thompson et al., 1994), and haplotypes were designated using DNASP version 6 (Rozas et al., 2017). The haplotype network was constructed using the Median Joining method in PopArt 1.7 (http://popart.otago.ac.nz) (Bandelt et al., 1999). Arlequin version 3.5.2.2 (Excoffier & Lischer, 2010) was used to estimate: (i) haplotype (*h*) and nucleotide diversity (π); (ii) Fu's Fs (Fu, 1997) and Tajima's D (Tajima, 1989) and test their significances following 10,000 permutations; and (iii) calculate pairwise *Φ*_ST_ with associated *p* values assessed by 10,000 permutations. Genetic structure was also assessed using Analysis of Molecular Variance (AMOVA) based on several groupings: (1) state/territory, (2) mainland × offshore, (3) mainland × islands (specifically grouping Rodrigues and Mauritius), (4) mainland × islands, and (5) regional groupings as per Van Nieuwenhove et al. (2019) (Table 2). Mismatch distribution, the frequency distribution of pairwise differences between haplotypes within a sample, and simulated distribution under a model of demographic expansion were compared using the sum of squared deviations (SSD) and Harpending's raggedness index (HRI) (Harpending, 1994) as a test statistic with significance assessed after 10,000 bootstrap replications (Felsenstein, 1985). Time since population expansion was estimated by *T = τ/2u* (Rogers and Harpending, 1992). Mutation rate (*u*) of 2% per million years based on the mutation rate of COI in cephalopods was used as levels of genetic variability for NCR (*ĥ* = 0.273 ± 0.143 SE) are comparable to levels of COI in *Octopus vulgaris* (Cuvier, 1797) (*ĥ* = 0.339 ± 0.213 SE) (Van Nieuwenhove et al., 2019) and *Octopus insularis* (Leite & Haimovici, 2008) (*h* = 0.210–0.483) (Lima et al., 2022), a proxy used in other Octopodidae studies (Pardo‐Gandarillas et al., 2018). Coalescent Bayesian skyline analysis was implemented in BEAST version 2.7.5 (Bouckaert et al., 2019) to produce estimates of effective population size (*N*_e_) through time and the highest posterior density intervals (95% HPD). The Hasegawa‐Kishino‐Yano substitution model (HKY) was identified as the best fit by the Bayesian information criterion (−lnL = 664.31, BIC = 1583.97) in jModelTest version 2.1.1 (Darriba et al., 2015). Since the analysis was performed on an intraspecies dataset, a strict clock model was selected as done in *Octopus hubbsorum* (S. S. Berry, 1953) (Dueñas‐Romero et al., 2020). Markov‐chain Monte Carlo (MCMC) was run using a single chain of 5 × 10^7^ iterations, sampling every 5000 generations; the first 5 × 10^6^ chains were discarded as burn‐in. Bayesian skyline plots were generated using Tracer version 1.7 (Rambaut, et al., 2018).

**TABLE 2:** Hierarchical AMOVA design of *O. cyanea* populations in the SWIO.

AMOVA design	*Φ*_CT_	*F*_CT_
Country	−0.005	0.000
KEN × TAN × MOZ × MAD × ROD × MAU × SEY		
African mainland × Offshore	−0.004	0.000
(KEN, TAN and MOZ) × (MAD, ROD, MAU, and SEY)		
African mainland × Islands based on the presence barrier currents	−0.004	−0.001
(KEN, TAN, and MOZ) × MAD × (ROD and MAU) × SEY		
Mainland v Islands	−0.002	0.001
(KEN, TAN and MOZ) × MAD × ROD × MAU × SEY		
Mainland & N MAD × KEN × SW MAD × MAU × ROD × SEY	−0.007	0.000
(TAN, MOZ, IV) × KEN × (AN, BE) × ROD × MAU × SEY		

*Note*: Country and territory are indicated as: KEN: Kenya, TAN: Tanzania, MOZ, Mozambique, MAD: Madagascar, ROD: Rodrigues, MAU: Mauritius, SEY: Seychelles, AN: Andavadoaka, BE: Beheloke, and IV: Ivovona. The Parentheses indicate grouped samples. Significant departure of values from zero is indicated by *p < .05.

#### 2.3.2 Analysis of microsatellite data

GENALEX 6.5 (Peakall & Smouse, 2006) assessed genetic variation within samples by several metrics: the number of alleles (*N*_A_), observed heterozygosity (*H*_O_), and expected heterozygosity (*H*_E_). Rarefied allelic richness (*A*_R_) (Hurlbert, 1971; Mousadik & Petit, 1996; Petit et al., 2008) was calculated in FSTAT version 2.9.4 (Goudet, 1995). GenePop version 4.7.5 (Rousset, 2008) was used to test for conformity of observed genotype frequencies to Hardy‐Weinberg equilibrium (HWE) expected proportions and genotypic linkage equilibrium between pairs of loci using exact tests incorporating a Markov chain algorithm (10,000 dememorization, 10,000 batches, 5000 iterations). Due to possible null allele effects indicated by heterozygote deficits, the software FreeNA (Chapuis & Estoup, 2007) was used to estimate global and pairwise *F*_ST_ values, applying the excluding null allele (ENA) correction. Pairwise ENA‐corrected *F*_ST_ values were visualized by Principal Co‐ordinates Analysis (PCoA) and inspected for isolation by distance using a Mantel test of correlation with the shortest ocean geographic distance in GENALEX. Pairwise geographic distances (km) were generated in Geographic Distance Matrix Generator version 1.2.3 (Ersts, 2011). AMOVA's were performed using the same groupings as for mtDNA.

Genetic structure was also assessed using the individual‐based Bayesian clustering method in STRUCTURE version 2.3 (Pritchard et al., 2000). The analysis was run for three iterations, assuming the user inferred *K* values ranging from 1 to 21 (total number of sampled sites +1). Each run had a burn in of 10^6^ steps and 10^6^ MCMC repetitions for each model. Model parameter combinations of admixture/no admixture and correlated/independent allele frequencies were varied over multiple analyses performed with and without prior sample knowledge (site location), as recommended by Hubisz et al. (2009). The most probable value of K was estimated using L(k) using the online tool Structure Harvester (Earl & vonHoldt, 2012; Evanno et al., 2005). This was complemented by classical Bayesian self‐assignment tests performed in GENALEX.

## 3 RESULTS

The final mitochondrial NCR dataset comprised 415 sequences (each of 437 bases) from 6 countries and the autonomous territory of Rodrigues in the SWIO, 206 of which were from individuals collected between 2010 and 12 across the SWIO, while the other 209 were collected exclusively from Tanzania in 2020 (Table 1). The microsatellite dataset consisted of 232 Tanzanian *O. cyanea* collected from 9 sites in 2020 and combined with a dataset of samples collected in 2010–12 spanning SWIO counties. The combined microsatellite dataset set included 962 individuals.

### 3.1 Mitochondrial analysis

**FIGURE 2:**
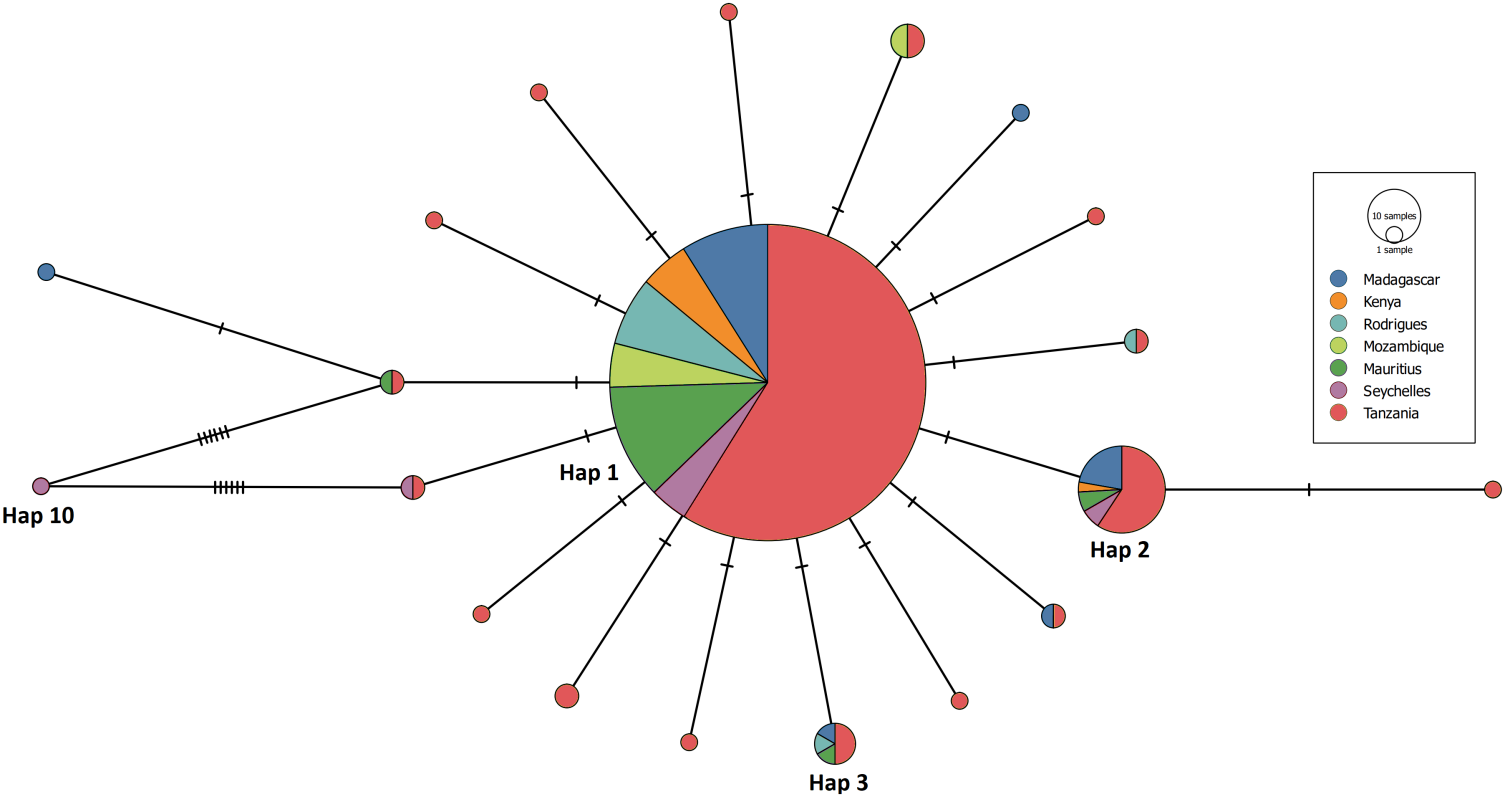
Median joining haplotype network of *O. cyanea* (*N* = 415) in the SWIO based on 437 bases of the mitochondrial NCR. Perpendicular dashes represent the number of mutations separating haplotypes, and circle size indicates the number of individuals with that haplotype. The Color represents the country of origin.

**FIGURE 3:**
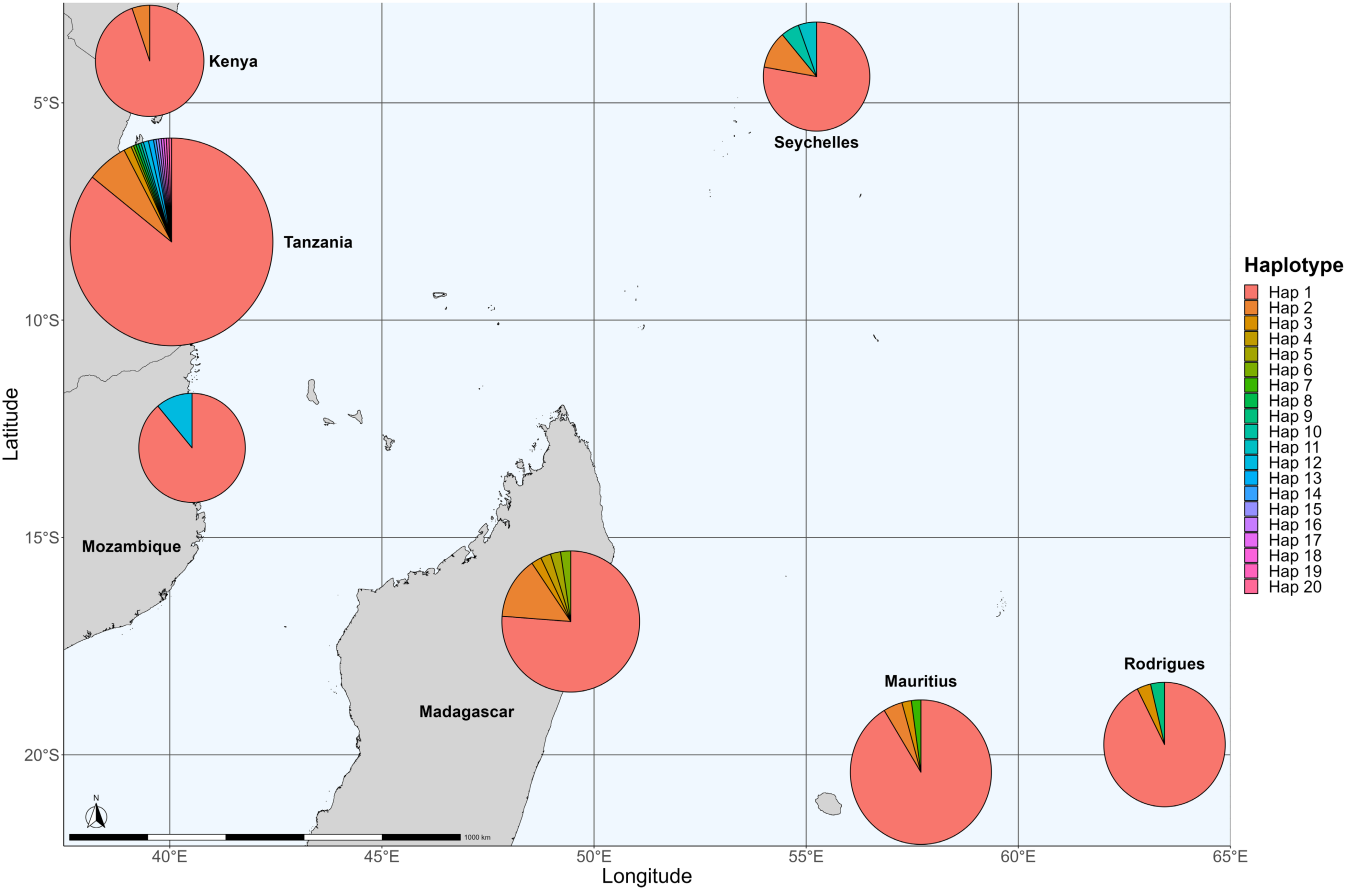
MtDNA NCR haplotype frequencies of *O. cyanea* (*N* = 415) across the SWIO. The radius of plots corresponds to the log_10_ of the total number of individuals sampled from a region.

From 415 individuals sequenced, 20 NCR haplotypes were resolved (GenBank accession PP448064–PP448083; Figure 2), defined by 19 transition and four transversion mutations. Haplotype 1 (357 individuals) was found to be ubiquitous across the SWIO, while haplotype 2 (found in 27 individuals) occurred in all sites but Rodrigues and Mozambique, and haplotype 3 (6 sequences) was found in low abundance in all countries except Kenya, Mozambique, and the Seychelles (Figure 3). Eleven private haplotypes were found: 8 in Tanzania, 2 in Madagascar, and 1 in the Seychelles. Haplotype 10 was observed in a single individual from the Seychelles and exhibited 6 mutational steps from other haplotypes identified in the Seychelles, Mauritius, and Tanzania.

**FIGURE 4:**
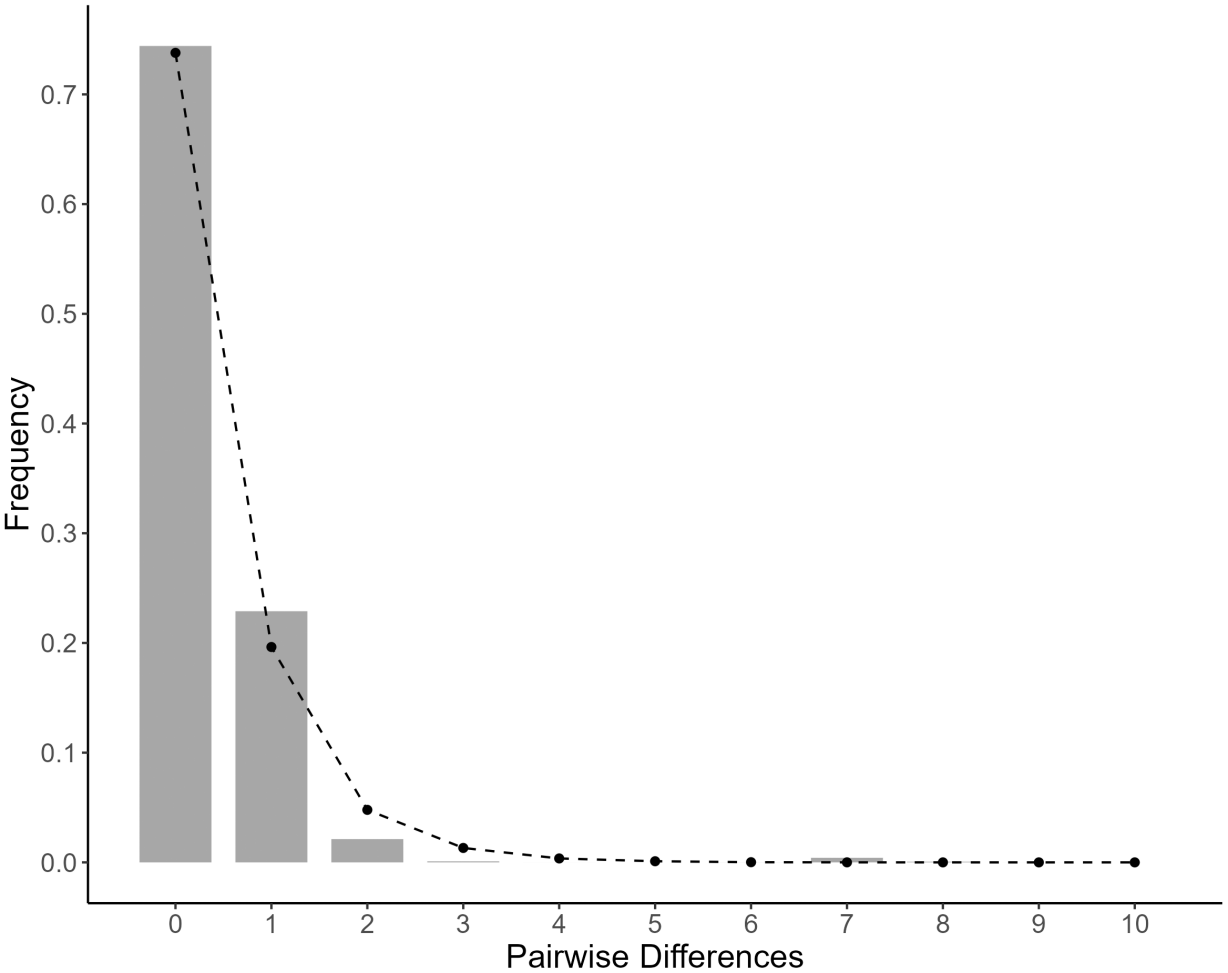
Mismatch distribution curves under a model of sudden demographic expansion for *O. cyanea* (*n* = 415) in the SWIO based on 437 bases of the mitochondrial NCR. Filled bars: observed frequency of pairwise distribution. Black dashed line: expected distribution. HRI: *r* = 0.309, *p* = .589; SSD = 0.002, *p* = .492.

**FIGURE 5:**
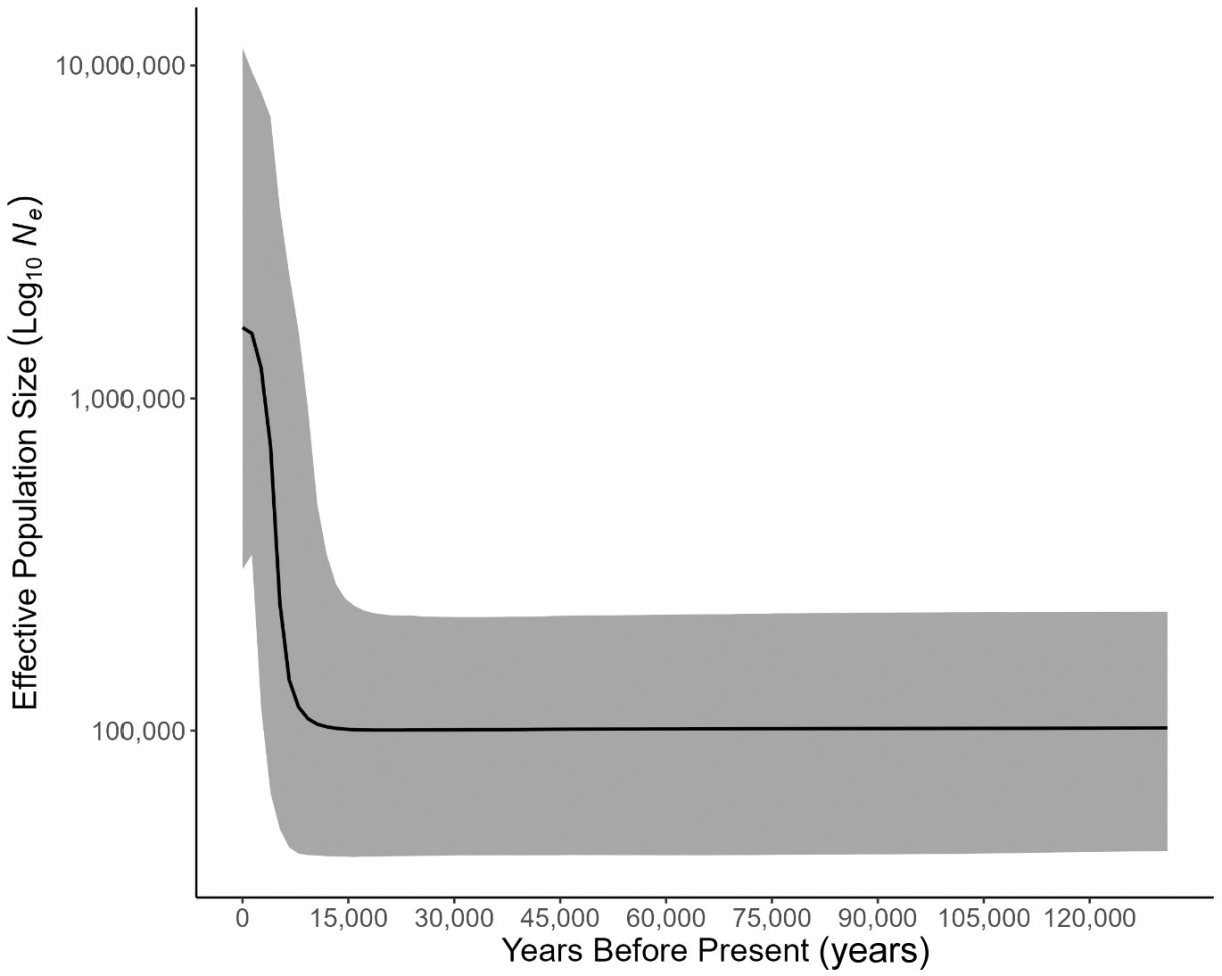
Bayesian skyline plot showing demographic change in effective population size of *O. cyanea* in the SWIO based on 437 bases of the mitochondrial NCR. The black line represents the mean log_10_ estimate of effective population size. The gray shaded area represents the 95% highest posterior density interval.

Haplotype diversity was found to range between 0.105 and 0.571 (average *h* = 0.273 ± 0.143 SE) at Mombasa and Kunduchi, respectively (Table 1). The number of haplotypes identified per sample ranged from 1 to 6, with Ivovona being the only site where a single haplotype was found and Pembeni being the only site with 6 haplotypes. Significant deviations from neutral expectations based on Tajima's D and/or Fu's Fs were found in at least one site within each state/territory, except for sites in Kenya, Mozambique, and Rodrigues (Table 1). Pooling samples by state/territory, Fu's Fs were significantly negative everywhere except Kenya, Mozambique, and the Seychelles, while Kenya and Mozambique were the only sites that did not report a significantly negative Tajima's D (Table S1). A mismatch distribution constructed using all 415 sequences displayed a unimodal peak (Figure 4). The hypothesis of sudden population expansion was not rejected for the overall pooled sample (HRI = 0.309, *p* = .589; SSD = 0.002, *p* = .492). The time since the expansion of *O. cyanea* in the SWIO was estimated at 171,700 years based on the value of *τ* = 3 and a substitution rate of 2% per million years. Bayesian skyline analysis indicated that the expansion of population size initiated ~10,000 YBP (Figure 5). Tests of genetic structure (*Φ*_ST_) at the NCR locus reported significant differentiation in 16 of 190 comparisons, with Kunduchi and Beheloke contributing 8 and 5 significant pairwise comparisons out of 16, respectively (Table 3). AMOVA reported nonsignificant inter‐group differentiation for all defined groupings, including management units suggested by Van Nieuwenhove et al. (2019) (*F*_CT_ = 0.011, *p* = .212). No temporal genetic variation was found between Tanzanian samples from 2010 to 2012 and 2020 (*F*_CT_ = 0.012, *p* = .146).

**TABLE 3:** Pairwise *Φ*_ST_ values for the mitochondrial noncoding region (top matrix) and *F*_ST_ values across 7 microsatellite markers following ENA correction (bottom matrix) among samples of *O. cyanea* from the SWIO.

	Kenya	Tanzania											Mozambique	Madagascar			Mauritius	Rodrigues		Seychelles
	MB	KU	ST	BW	FU	JI	JO	KW	MG	MS	MT	PM	PE	AN	BE	IV	MH	NR	SR	PR
MB	‐	0.103	−0.036	−0.026	−0.033	0.009	−0.065	−0.036	−0.002	−0.003	−0.031	−0.027	0.041	0.008	0.041	−0.055	−0.031	0.001	0.011	0.005
KU	0.003	‐	0.108*	0.069	0.145*	−0.013	0.031	0.090*	0.158*	0.178**	0.078	0.064	0.151**	0.001	−0.027	0.088	0.152**	0.145*	0.110	0.005
ST	0.003	0.005	‐	−0.017	−0.019	0.003	−0.047	−0.021	0.000	−0.021	−0.019	−0.017	0.033	−0.011	0.051	−0.058	−0.028	−0.041	0.000	0.013
BW	0.004	0.007	0.006	‐	−0.029	0.003	−0.046	−0.016	0.003	0.007	−0.016	−0.016	−0.022	−0.006	0.031	−0.054	−0.005	−0.003	−0.010	0.001
FU	0.003	0.00	0.002	−0.003	‐	0.038	−0.037	−0.014	0.001	0.000	−0.012	−0.010	−0.004	0.032	0.075*	−0.061	−0.015	−0.002	0.007	0.028
JI	0.010	0.009	−0.001	0.009	0.004	‐	−0.036	0.005	0.060*	0.058*	0.002	0.001	0.067	−0.029	−0.026	0.003	0.035	0.033	0.033	−0.005
JO	0.004	0.002	0.002	0.001	0.003	0.001	‐	−0.055	−0.002	0.002	−0.051	−0.046	0.030	−0.031	−0.010	−0.037	−0.039	0.003	0.000	−0.026
KW	0.004	0.007	0.005	−0.001	0.006	0.004	−0.001	‐	0.013	0.014	−0.031	−0.016	0.042	0.012	0.034	−0.044	−0.013	0.008	0.009	0.016
MG	0.008	0.011	0.006	−0.004	0.003	−0.007	0.007	0.006	‐	0.001	0.009	0.004	0.033	0.035	0.089*	−0.058	0.006	−0.003	−0.048	0.029*
MS	0.001	0.003	0.004	−0.002	0.002	−0.001	−0.009	0.002	−0.001	‐	0.011	0.007	0.038	0.026	0.109**	−0.061	−0.005	−0.037	0.005	0.023
MT	0.002	0.005	0.003	−0.003	−0.003	−0.001	−0.001	−0.002	0.000	−0.002	‐	−0.014	0.032	0.006	0.030	−0.048	−0.009	0.002	−0.001	0.013
PM	0.012	0.017	0.011	0.009	0.004	0.004	0.014	0.015	0.006	0.009	0.000	‐	0.020	−0.002	0.026	−0.053	−0.004	−0.005	−0.012	0.011
PE	0.004	0.007	0.001	0.002	0.001	−0.002	0.003	0.000	0.002	0.004	−0.001	0.010	‐	0.038	0.102*	−0.007	0.043	0.035	0.029	0.029
AN	0.002	0.005	0.000	0.002	0.001	0.000	0.004	0.003	0.004	0.003	0.001	0.004	0.002	‐	0.008	−0.032	0.017	−0.007	0.000	−0.026
BE	0.004	0.008	0.001	0.006	0.001	−0.004	0.004	0.006	0.001	0.004	−0.001	0.003	0.001	−0.002	‐	0.041	0.081*	0.089	0.069	0.010
IV	0.000	0.001	0.002	0.004	0.002	0.002	−0.003	0.002	0.005	−0.001	0.000	0.011	0.003	0.001	0.000	‐	−0.057	−0.050	−0.032	−0.035
MH	0.006	0.010	0.006	0.009	0.010	0.008	0.009	0.005	0.010	0.007	0.008	0.016	0.006	0.007	0.008	0.004	‐	−0.017	0.008	0.040
NR	0.001	0.002	0.001	0.003	0.005	0.004	−0.001	0.003	0.009	0.002	0.003	0.018	0.003	0.001	0.002	0.001	**0.007**	‐	0.006	0.015
SR	−0.002	0.004	0.003	0.001	0.001	0.007	0.006	0.001	0.003	0.003	−0.001	0.005	0.003	−0.001	0.001	0.001	0.009	0.000	‐	−0.005
PR	0.006	0.003	0.004	0.005	0.005	0.003	0.000	0.005	0.006	0.003	0.004	0.011	0.003	0.003	0.004	0.002	**0.008**	0.003	0.003	‐

*Note*: In the lower diagonal, underlined values are significantly greater than zero following the ENA correction (95% bootstrap). Both analyses follow 10,000 permutations. Significant departure of values from zero is indicated by *p < .05 and **p < .01 in the top diagonal.

### 3.2 Microsatellite analysis

Summary indices are presented in Table 1. Of 147 locus by sample Exact Tests performed on genotype frequencies, 68 deviated from Hardy‐Weinberg expectations, all due to heterozygote deficits. No significant genotypic linkage disequilibrium was detected between any pair of loci in global tests. The total number of alleles per locus across sampling sites ranged from 4 (OC22) to 19 (ROC6 and OC18). All sites exhibited similar values for allelic richness (range 9.74 (±0.82 SE) to 11.08 (±0.89 SE)) and expected heterozygosity (range 0.79 (±0.07 SE) to 0.86 (±0.03 SE)). Observed heterozygosity was found to vary more widely among sites, ranging from 0.51 (±0.10 SE) to 0.81 (±0.07 SE). In all samples, mean *H*_E_ was greater than *H*_O_, resulting in positive *F*_IS_ values between 0.033 and 0.376, with 19 out of 20 values being significantly greater than expectations (Table 1).

**FIGURE 6:**
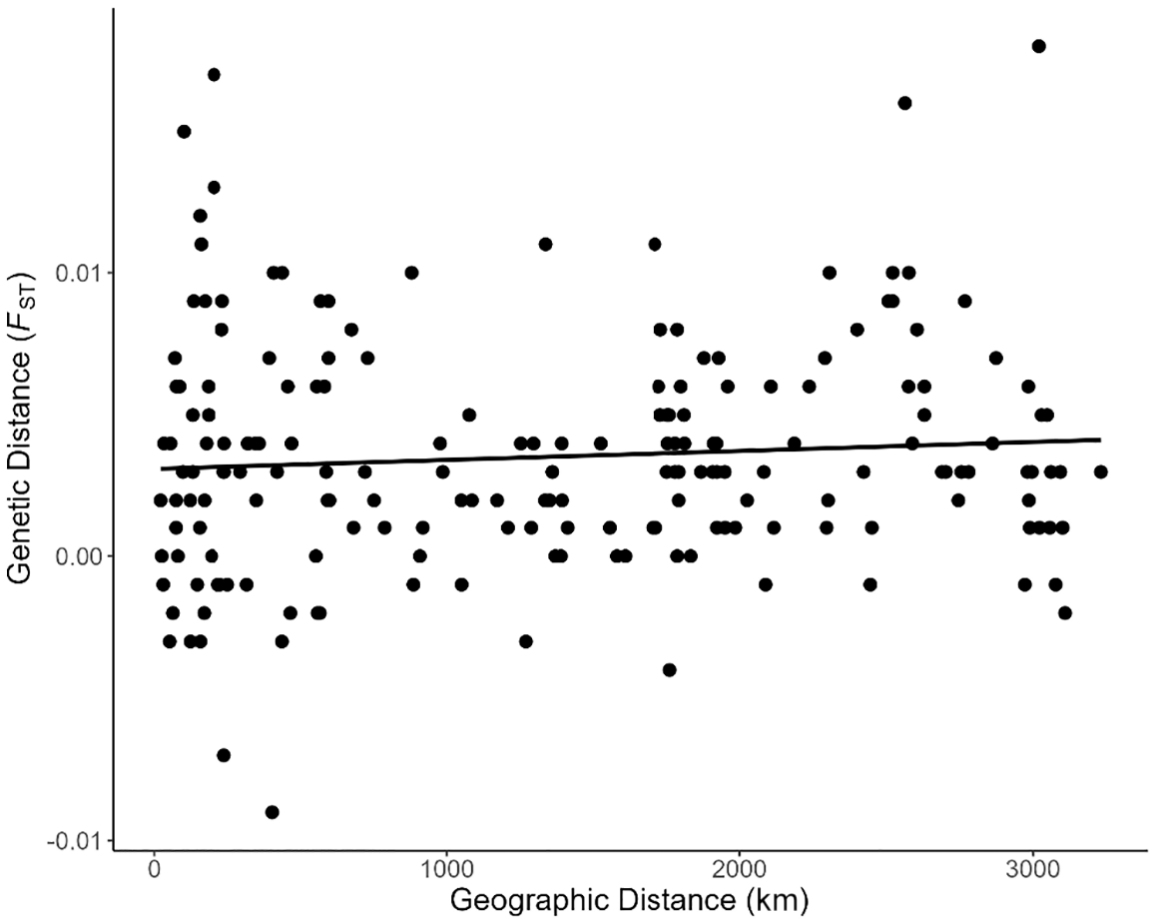
Mantel test for correlation between geographical distance (km) and microsatellite genetic difference (*F*_ST_) between all pairwise sample comparisons (*R* ^2^ = .006, *p* = .282).

**FIGURE 7:**
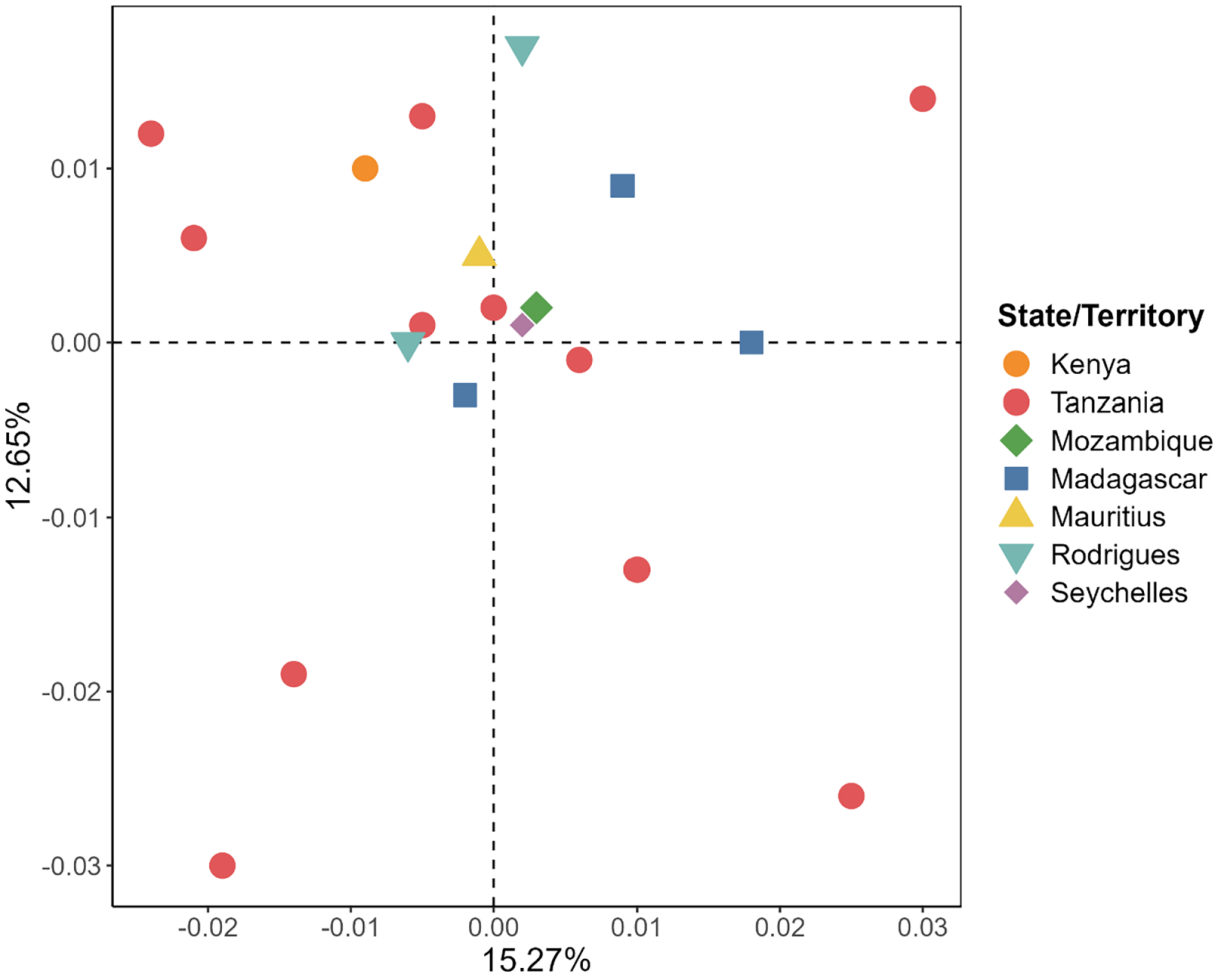
Principal co‐ordinate analysis (first two axes) of pairwise microsatellite *F*_ST_ values of *O. cyanea* samples from 20 sites in the SWIO. Color‐coded symbols represent the state/territory of origin.

Across all samples, global microsatellite differentiation was statistically significant but numerically very small (*F*_ST_ = 0.004, *p* = .005), with 43 of 190 ENA‐corrected pairwise tests being significant (Table 3). Of the pairwise *F*_ST_ tests, the Kunduchi and Pembeni samples represented 10 and 9 significant results of 43, respectively, post null allele correction. There was no significant correlation between the observed pairwise *F*_ST_ values (ENA‐corrected) and geographic distance (*R* ^2^ = .006, *p* = .282 – Figure 6), with a lack of any geographical pattern also obvious from PCoA (Figure 7). Temporal genetic variation between Tanzanian samples from 2010 to 2012 and 2020 was nonsignificant (*F*_CT_ = 0.004, *p* = .167). There were no cases of significant variation between groups in any AMOVA design, including between the management units suggested by Van Nieuwenhove et al. (2019): *F*_CT_ = <0.001, *p* = .413. The Bayesian clustering analysis produced a model of *K* = 1, and in agreement with this suggested lack of individual‐based structure, assignment tests found low rates of self‐assignment when samples were grouped by state/territory (7 groups: 23%) or treated independently (20 groups: 8%).

## 4 DISCUSSION

The present study combined mitochondrial DNA and nuclear microsatellite DNA markers to assess the degree of spatial genetic structuring and diversity of *O. cyanea* populations throughout the SWIO. The mtDNA data revealed a high diversity of haplotypes sharing a shallow phylogeny across the region, with additional signals of historic population size fluctuations supported by demographic tests. *F*_ST_ and Bayesian clustering analyses of microsatellite data identified an absence of clear genetic structure across the region, with a low level of significant “patchy” differentiation. While pairwise *F*_ST_ tests did result in a number of significant differences, in all cases, the values were low (all <0.018, all but 13 tests below 0.010). They exhibited no obvious geographic pattern, association with oceanographic features (e.g. island versus mainland systems), or signal of Isolation‐By‐Distance. Overall, our findings indicate extensive gene flow and connectivity in *O. cyanea* populations throughout the SWIO.

### 4.1 Phylogeographic structure and population history

The mtDNA data, showing a star‐shaped haplotype network and shallow phylogeny (Slatkin & Hudson, 1991), with demographic tests indicating historical population size fluctuations, are in agreement with population demographic patterns reported by Van Nieuwenhove et al. (2019). Van Nieuwenhove et al. (2019) attributed such population fluctuations to habitat changes during Pleistocene glacial and interglacial periods. During the Pleistocene epoch, sea levels were ~ 120 m lower than present day on at least 3 occasions (250,000, 150,000, and 17,000 years ago) and experienced gradual change governed by climate during the thousands of years preceding (Muths, Tessier, et al., 2011). The gradual change in sea level was likely accompanied by a reduction in available coastal habitat for shallow benthic species due to the steep gradient of the East African shelf edge, resulting in recurrent population bottlenecks (Gross et al., 1982; Green, 2009). This was followed by a period of recolonization and demographic expansion, with sea level rising in the ensuing interglacial periods. Mismatch distribution analysis suggested an estimated time of *O. cyanea* population expansion of ~171,700 YBP. However, if a 10‐fold mutation rate correction is applied as proposed by previous studies (González‐Wevar, David, & Poulin, 2011; Pardo‐Gandarillas, Ibáñez, Yamashiro, Méndez, & Poulin, 2018; Mckeown, Watson, Coscia, Wootton, & Ironside, 2019; Healey et al., 2020), the time of expansion is estimated to be ~17,000 YBP. This rate‐corrected estimate is supported by Bayesian skyline analysis, suggesting the onset of demographic expansion occurring ~10,000 YBP. While these estimates are not precisely congruent with one another, they roughly correspond to a post LGM expansion. Regardless of the exact timing, eustatic sea level fluctuations during the Pleistocene are proposed to have effected demographic changes in *O. cyanea* as observed across the SWIO region for a number of marine fish (Craig, Eble, Bowen, & Robertson, 2007; Visram et al., 2010; Healey et al., 2017, 2020) and crustacean species (Tolley, Groeneveld, Gopal, & Matthee, 2005; Gopal, Tolley, Groeneveld, & Matthee, 2006; Fratini, Ragionieri, & Cannicci, 2010). The lack of phylogeographic structuring within the *O. cyanea* mtDNA data is compatible with a lack of prolonged population vicariance across the SWIO. Similar shallow phylogenies have been reported in many other SWIO species, and so it would appear that for many species there has been little phylogeographic diversification within the SWIO (Hoareau, Boissin, & Berrebi, 2012; Muths, Tessier, & Bourjea, 2015), with the majority of cases of deep phylogeographic structure within the region due to colonization of allochthonous lineages (Ragionieri, Fratini, Vannini, & Schubart, 2009; Ragionieri, Cannicci, Schubart, & Fratini, 2010; Silva, Mesquita, & Paula, 2010).

### 4.2 High gene flow across the SWIO

Pairwise *Φ*_ST_/*F*_ST_ values from mtDNA and microsatellite data were numerically small and mostly nonsignificant, while individual‐based analyses did not reveal any clusters overall, indicating a lack of genetic differentiation and thus suggesting high gene flow within the studied area. The pairwise *Φ*_ST_ values here are consistent with values in the order of 0.01 reported by Van Nieuwenhove et al. (2019), where 89.1% of pairwise tests were found to be nonsignificant. Given that *O. cyanea* adults are sedentary, this connectivity is likely driven by paralarval dispersal. The species planktonic larval duration (PLD) is approximately 30 days, varying with temperature (Guard & Mgaya, 2002). Such a PLD in an ocean region where marine currents typically range from 20 to 30 Sverdrup (~0.11 m s^−1^) and vary in direction and intensity during the southern winter (including reversal of the North flowing Somali current) would facilitate passive long‐distance dispersal of paralarvae over hundreds of kilometers (Ali & Huber, 2010; Schott, Xie, & McCreary, 2009). The lack of genetic structure reported for *O. cyanea* fits with the general pattern of geographically extensive gene flow reported for other cephalopod species with planktotrophic paralarval dispersal such as *Loligo forbesii* (Steenstrup, 1856) (Shaw, Pierce, & Boyle, 1999), *Doryteuthis opalescens* (S. S. Berry, 1911) (Reichow & Smith, 2001), *Loligo reynaudii* (d'Orbigny,1839–1841) (Shaw et al., 2010), *Doryteuthis pealeii* (Lesueur, 1821) (Shaw et al., 2010), and *Doryteuthis gahi* (d'Orbigny, 1835) (McKeown, Arkhipkin, & Shaw, 2019). Spatial genetic structuring in cephalopods seems to occur where there is some form of oceanographic/physical barrier to dispersal (Sandoval‐Castellanos, Uribe‐Alcocer, & Díaz‐Jaimes, 2007; Staaf et al., 2010; McKeown, Arkhipkin, & Shaw, 2019), and there are few (if any) major discrete hydrodynamic barriers in the WIO area covered by the present study.

All *O. cyanea* samples were found to exhibit significant deficits of heterozygotes. In their SNP‐based study of *Illex argentinus*, Chemshirova et al. (2023) described significant heterozygote deficits for all samples within a panmictic population. The authors attributed this to intra‐annual pulses of recruitment generating ephemeral genetic differences among groups, mostly likely at the larval, early recruit stage, followed by mixing at later life history stages. Heterozygote deficits among adult samples of *I. argentinus* had previously been described by Adcock et al. (1999), with the authors excluding inbreeding or mixing of genetically distinct populations as causes. Similar patterns have also been reported in Adriatic Sea species of cephalopods by Garoia et al. (2004), which the authors linked to spawning at different times. In addition to generating heterozygote deficits within populations, such processes of pulsed and variable recruitment are likely to also drive genetic patchiness among areas, as described by Cheng et al. (2021), who concluded that intra‐annual recruitment pulses underpinned a low level of local patchy structure despite no macrogeographical structure in *D. opalescens*. Similar recruitment processes could explain the patchy, unstructured differentiation among the 43 significant pairwise tests reported here. Post settlement, *O. cyanea* becomes resident in their local habitat (~50 m), only moving to new dens after three to five weeks in some adults (Yarnall, 1969; Van Nieuwenhove, Ratsimbazafy, & Kochzius, 2019). It is noteworthy that the sedentary nature of *O. cyanea* would be more conducive to the persistence of such signatures of larval recruitment heterogeneity compared to other species with greater postlarval dispersal (Healey et al., 2017; McKeown et al., 2017; McKeown, Taylor, & Shaw, 2018; Mckeown, Watson, Coscia, Wootton, & Ironside, 2019; Planes & Lenfant, 2002).

### 4.3 Levels of genetic variation compared to other cephalopods

Genetic variability is crucial for maintaining sustainable yields and population adaptability (Kenchington, Heino, & Nielsen, 2003). Levels of intrasample genetic diversity were similar across all samples of *O. cyanea*, providing no evidence of reduced genetic variation among any spawning group. Overall levels of nuclear diversity (*N*_A_ = 10.29–16.43, *A*_r_ = 9.74–11.08, *H*_E_ = 0.792–0.855, *H*_O_ = 0.510–0.809) were found to be similar to those of *Octopus minor* (Sasaki, 1920) (*N*_A_ = 8.9–12.1, *A*_r_ = 7.9–10.3, *H*_E_ = 0.727–0.766, *H*_O_ = 0.534–0.669; Kang et al., 2012), *Octopus maya* (G. L. Voss & Solís, 1966) (*N*_A_ = 6.4–9.8, *H*_E_ = 0.62–0.64, *H*_O_ = 0.60–0.64; López‐Galindo et al., 2018), and *O. vulgaris* (*N*_A_ = 3–31, *A*_r_ = 1.36–15.65, *H*_E_ = 0.06–0.92, *H*_O_ = 0.04–0.89; Pirhadi et al., 2023). Levels of mtDNA NCR diversity in *O. cyanea* (*ĥ* = 0.273 ± 0.143 SE, π = 0.0002–0.0025) were also similar to levels reported for mtDNA COI in the SWIO by Van Nieuwenhove et al. (2019) (*h* = 0–0.71, *ĥ* = 0.339 ± 0.213 SE, π = 0–0.0015), and other octopus species analyzed at the COI gene: *O. vulgaris* (*h* = 0–0.83, *ĥ* = 0.355 ± 0.365 SE, π = 0–0.38; Van Nieuwenhove et al., 2019); *O. minor* (*h* = 0.398–0.888, π = 0.0005–0.0058; Xu et al., 2018) and *O. insularis* (*h* = 0.210–0.483, π = 0.0089–0.0019; Lima et al., 2022). The data therefore support the view that, despite harvesting intensity and previously mentioned recruitment variability in *O. cyanea*, if current stock sizes are maintained, genetic drift is insufficient to reduce genetic variation. Similarly, Adcock et al. (1999) previously demonstrated that *I. argentinus* maintained high levels of intrasample diversity even during periods of intense harvesting pressure.

The control region (CR)/Noncoding region (NCR) harbors the regulatory elements required for the replication and expression of the mitochondrial genome (Shadel & Clayton, 1997). However, as it does not code for a functional gene, it is typically expected to accumulate mutations more rapidly than other mtDNA regions, with this conferring high investigative power for population and phylogeographic analyses (Shadel & Clayton, 1997). The majority of investigations of CR/NCR divergence among marine invertebrates have focused on decapod crustaceans (Chu, Li, Tam, & Lavery, 2003; Diniz, Maclean, Ogawa, Cintra, & Bentzen, 2005; McMillen‐Jackson & Bert, 2003; McMillen‐Jackson & Bert, 2004), with only a handful of investigations assessing NCR variation in cephalopods (Aoki, Imai, Naruse, & Ikeda, 2008; Winkelmann et al., 2013). A comparison of NCR data from this study and COI data from Van Nieuwenhove et al. (2019) revealed nearly identical levels of variation, contrary to our prior expectation that the NCR may harbor higher levels of diversity. This aligns with other studies that have found low mutation accumulation rates at NCRs in other cephalopods (Aoki, Imai, Naruse, & Ikeda, 2008; Winkelmann et al., 2013). While interspecific data would be useful to test for factors such as hotspot saturation (Galtier, Enard, Radondy, Bazin, & Belkhir, 2006; Alter & Palumbi, 2009) or selective constraints, the data here indicate that the NCR is not a hypervariable region in this species.

### 4.4 Implications for management and further research

It is important to note that levels of gene flow sufficient to limit population differentiation may fall short of the dispersal required to replenish harvested stocks (Hauser & Carvalho, 2008). Therefore, despite the low levels of genetic structure observed in the SWIO being compatible with high gene flow, there may be some contemporary independence of stocks significant for management. An additional consideration here is that the mtDNA results suggest that populations may not be at migration drift equilibrium. Contemporary connectivity may therefore be overestimated due to historical gene flow and genetic inertia. For these reasons, the resolution of spatial stock structure may be beyond the resolution of neutral genetic markers and would benefit from complementary analysis of markers under selection (Canino, O'Reilly, Hauser, & Bentzen, 2005). Future studies should therefore consider the analysis of genome‐wide single nucleotide polymorphisms (SNPs) as these markers have already been shown to improve estimates of population and demographic parameters in other exploited cephalopods (Cheng et al., 2021). Such genomic analyses should be combined with studies of paralarval movement to identify and protect critical habitats, known as octopus replenishing zones. A deeper understanding of the spatial and temporal abundances of paralarvae could also direct dynamic management strategies. For example, the timing of seasonal closures could be aligned with critical times of the year when paralarval dispersal is more active.

Establishing management units that coincide with biological populations is critical for the sustainable management of a species (Reiss, Hoarau, Dickey‐Collas, & Wolff, 2009). Monitoring efforts will be compromised if management units encompass only a fraction of a broader population (Frisk, Miller, Martell, & Sosebee, 2008), while management units including multiple populations may also lead to imprecise estimates of population‐specific abundances and productivity, with the potential to mask declines in vulnerable populations (Kell et al., 2009; Ying, Chen, Lin, & Gao, 2011). Regional fishing communities of the SWIO have already established a range of restrictions on the octopus fishery to ensure sustainability and economic security, including rotational closures, seasonal closures, size limits, gear limits, and licensing (Chande, Mgaya, Benno, & Limbu, 2021; Jhangeer‐Khan, Agathe, & Yvergniaux, 2015; Sauer et al., 2021). Community‐focused fishery closures have positive impacts on local economies and octopus stocks, leading to increased yields and greater incomes surpassing the costs of closure (Oliver et al., 2015; Silas et al., 2022). Since the results of this study indicate high connectivity of *O. cyanea* throughout the SWIO, fishery closures and other measures that affect their populations should be coordinated across geopolitical boundaries. The Collaborative Fisheries Management Areas in Tanzania represent an operational model that could be extended to other areas. This should also be part of a broader adaptive management strategy to address changes in ocean temperatures, currents, and other environmental variables that have an impact on paralarval dispersal patterns and to educate communities about the importance of protecting octopus species paralarvae.

## AUTHOR CONTRIBUTIONS

**Charles R. Treleven:** Writing – original draft (equal); writing – review and editing (equal). **Mary A Kishe:** Data curation (equal); funding acquisition (equal). **Mathew O. Silas:** Investigation (equal). **Benjamin P. Ngatunga:** Writing – review and editing (equal). **Bigeyo N. Kuboja:** Funding acquisition (equal); writing – review and editing (equal). **Said S. Mgeleka:** Investigation (equal). **Amy L. Taylor:** Data curation (equal). **Megan A. M. Elsmore:** Methodology (equal). **Amy J. E. Healey:** Methodology (equal). **Warwick H. H. Sauer:** Funding acquisition (supporting); project administration (supporting). **Paul W. Shaw:** Funding acquisition (supporting); project administration (equal); writing – original draft (supporting). **Niall J. Mckeown:** Formal analysis (equal); writing – original draft (equal); writing – review and editing (lead).

## FUNDING INFORMATION

The authors would like to acknowledge part funding support for this work from the World Bank‐funded SWIOfish programme and the EU‐funded ReCoMaP programme.

## CONFLICT OF INTEREST STATEMENT

The authors declare no conflict of interest.

## Supporting information


Table S1

## Data Availability

All data are publicly available from pure.aber.ac.uk. Sequence data are also available from GenBank (PP448064–PP448083).
