# Supplementary material for: Genetic analysis of Octopus cyanea reveals high gene flow in the South‐West Indian Ocean
Source: Ecol Evol. 2024 Apr 4;14(4):e11205. doi: 10.1002/ece3.11205 (PMC10994983; doi:10.1002/ece3.11205)
Supplement: Supplementary file 1 — Table S1 [file ECE3-14-e11205-s001.docx]

**Supplementary Material**

Table 1: Summary information for *O. cyanea* samples from the SWIO using the mitochondrial noncoding region (*N* = 415) and 7 microsatellite markers (*N* = 962): sample size (*n*), number of haplotypes (*N_hap_*), haplotype diversity *(h*), nucleotide diversity (*π*), Fu’s Fs test (*F_S_*), Tajima’s D test (*D*), mean number of alleles (*N_A_*), rarified allelic richness (15 diploid individuals) (*A_R_*), observed heterozygosity (*H_O_*), expected heterozygosity (*H_E_*), and multilocus inbreeding coefficient values (*F_IS_*). Significant departure of values from expectations is indicated by * = *P*<0.05, ** = *P*<0.01, and *** = *P*<0.001.

|  | Mitochondrial | | | | | | Microsatellite | | | | | |
| --- | --- | --- | --- | --- | --- | --- | --- | --- | --- | --- | --- | --- |
| State/territory | *n* | *N_hap_* | *h* | *π* | *F_S_* | *D* | *n* | *N_A_* | *A_R_* | *H_O_* | *H_E_* | *F_IS_* |
| Kenya | 19 | 2 | 0.105 | 0.000 | -0.838 | -1.165 | 57 | 14.429 | 14.164 | 0.749 | 0.810 | **0.084*** |
| Tanzania | 245 | 17 | 0.262 | 0.000 | **-27.426***** | **-2.212***** | 370 | 19.714 | 14.683 | 0.672 | 0.834 | **0.194*** |
| Mozambique | 18 | 2 | 0.209 | 0.000 | -0.011 | -0.529 | 52 | 15 | 14.841 | 0.730 | 0.830 | **0.127*** |
| Madagascar | 42 | 6 | 0.407 | 0.001 | **-3.556**** | **-1.714*** | 210 | 18 | 14.696 | 0.773 | 0.847 | **0.080*** |
| Mauritius | 46 | 4 | 0.167 | 0.000 | **-3.432**** | **-1.576*** | 75 | 15.429 | 14.285 | 0.770 | 0.822 | **0.071*** |
| Rodrigues | 27 | 3 | 0.145 | 0.000 | **-2.223**** | **-1.512*** | 91 | 15.714 | 14.515 | 0.763 | 0.830 | **0.080*** |
| Seychelles | 18 | 4 | 0.399 | 0.002 | 0.062 | **-1.854*** | 107 | 16.429 | 14.788 | 0.786 | 0.840 | **0.072*** |
